# Potential use of DNA methylation in cervical swabs for early ovarian cancer diagnosis

**DOI:** 10.1186/s13048-025-01609-2

**Published:** 2025-02-15

**Authors:** Edyta Biskup, Joanna Lopacinska-Jørgensen, Claus Høgdall, Estrid V. Høgdall

**Affiliations:** 1 Department of Pathology, Herlev Hospital, University of Copenhagen, Herlev, Denmark; 2 Department of Gynaecology, Juliane Marie Centre, Rigshospitalet, University of Copenhagen, Copenhagen, Denmark

**Keywords:** DNA methylation, Ovarian cancer, Early diagnosis, Cervical swabs

## Abstract

**Introduction:**

Early diagnosis of ovarian cancer, using cost-effective and non-invasive methods remains an unmet medical need, largely due to unspecific symptoms of the disease.

**Objective:**

Our goal was to identify differentially methylated CpG loci between cervical swabs obtained from patients diagnosed with benign ovarian disease and with malignant pelvic mass.

**Methodology:**

Using Infinium EPICv2 array, we interrogated methylation profiles of 77 cervical swabs. The study cohort was then divided into a training and testing set to develop a diagnostic signature. We applied several strategies to pinpoint CpG sites able to differentiate cervical swabs obtained from ovarian cancer patients and patients with benign ovarian disease.

**Results and conclusions:**

None of the statistical methods applied identified CpG loci capable of diagnosing ovarian cancer with sufficient specificity and sensitivity. We conclude that methylation differences observed do not adequately distinguish between benign and malignant ovarian disease. The variations attributable to clinical conditions are likely obscured by the differences in cell composition, which is the primary source of sample heterogeneity. Therefore, we suggest that diagnostic tools should not rely on local methylation profile of the cervix but rather focus on detecting cancer-specific sequences transferred from the tumor site and present in cervical swabs.

**Non-technical summary:**

Ovarian cancer is difficult to detect early, and we aimed to explore whether DNA methylation in cervical swabs could serve as a diagnostic tool. However, our study found that methylation patterns in these samples do not reliably distinguish between benign and malignant conditions, likely due to variations in cell composition. We recommend future research focus on detecting tumor-specific DNA sequences in cervical swabs instead.

**Supplementary Information:**

The online version contains supplementary material available at 10.1186/s13048-025-01609-2.

## Introduction

Identifying biomarkers that would support early detection of ovarian cancer (OC) is still an unmet medical need, as most OC cases are diagnosed at late stages, where 5-year survival is approx. 30%. So far, no biomarker has been proven optimal for screening. This can be ascribed to unspecified early symptoms and lack of specific biomarkers [1].

Malignant ovarian tumors derive in over 90% cases from the epithelium and can be divided into two types based on their grade. Type I tumors, including low-grade serous tumors, mucinous, endometrioid, and clear cell carcinomas, progress relatively slowly. In contrast, Type II tumors, such as high-grade serous ovarian carcinoma (HGSOC), exhibit a more aggressive phenotype. HGSOC is also the most prevalent subtype [2].

OC may originate in the ovary itself or from neighboring structures, usually the distal fallopian tube [3]. In approximately 8% OC cases malignant neoplasms have an extraovarian origin, most commonly metastasizing from colorectum, stomach, breast and uterus [4].

Previously, we successfully identified 21 DNA positions which methylation levels differed between benign/borderline tumors and HGSOC [4]. However, this type of material can only be obtained during surgical intervention and the latter is only performed if the presence of pelvic mass has already been detected. Thus, ovarian tissue samples, albeit a source of information about tumor biology and underlying disease mechanisms, cannot possibly be used for early diagnosis. At the same time, using surrogate material, such as cervical swabs, is an attractive option, as this type of material is easily available and can be obtained in a non-invasive way. Notably, cervical swabs are already routinely collected for cervical cancer screening and provide an existing framework that could potentially be adapted for early OC diagnosis [5].

Several studies suggested possible use of cervical swabs for early diagnosis of OC, either focusing on detection of DNA mutations [6–11] or DNA methylation [12, 13]. All these studies build on an assumption that tumor cells shed from the ovaries down the reproductive tract to the cervical canal, and there the tumor DNA can be detected in a minimally invasive manner. Barrett et al. postulated that the DNA methylome of cervical cells differ between OC patients and healthy controls, and those changes are not driven by the presence of tumor DNA, but rather by an epigenetic differentiation defect [14]. They also devised a signature, named WID-OC index, which would allow identifying woman in the risk of cancer, based on methylation levels of 14,000 CpGs. Here, we evaluated if the extent of differences in the DNA methylomes between OC patients and women with benign ovarian disease would enable a design of a diagnostic panel, utilizing cervical swabs for early detection of OC. Our study builds on the assumption by Barrett and colleagues, analyzing the full methylation landscape of cervical swabs, while considering the hypothesis that differences in these profiles are not caused by tumor DNA shed from the ovaries.

## Methods

### Patient samples

We used two types of samples: 72 (70) ovarian tissue samples, which served as a reference and were used for comparative purposes, and 92 (77) cervical swabs; numbers in brackets refer to the number of samples which passed quality check and were used for the analyses. Samples from 14 patients were present in both groups.

The same ovarian tissue sample cohort has also been used in our previous study, where it has been characterized in detail with respect to cell composition [15]. It also partly overlaps with the cohort used for identification of CpG sites specific for HGSOC [4]. Both cohorts (tissue and swabs) consisted of samples collected from patients admitted to Gynecologic Clinic at Rigshospitalet (Copenhagen, Denmark) included in the Pelvic Mass/GOVEC study. Each cohort contained three types of samples: (i) benign/borderline (ii) HGSOC (iii) “other”, which was a mixed group containing OC subtypes other than HGSOC and ovarian malignancies of extraovarian origin. Patient clinical characteristics can be found in Table 1).

**Table 1:** Clinicopathological features. Data was retrieved from the Danish Gynecological Cancer database (DGCD; www.dgcg.dk/) register. Other– OC subtypes other than HGSOC and cases when the malignant mass is present in ovaries, but with extraovarian primary site. Numbers in brackets refer to the actual number of samples used in the analyses, in case some of the samples failed quality check

**Ovarian tissue samples**			
	**Benign/borderline**	**HGSOC**	**Other**
**No. of cases**	*N* = 19 (17)	*N* = 37	*N* = 16
**Median age in years (range)**	57.53 (20.5–86.2)	65.7 (41.1–84.1)	61.6 (31.9–83.4)
**FIGO Stage**			
**I**	4 (3)	1	1
**II**	1	6	4
**III**	1	30	4
**IV**			3
**NA**	13 (12)		4
**Cervical swabs**			
	**Benign/borderline**	**HGSOC**	**Other**
**No. of cases**	38 (34)	29 (24)	25 (19)
**Median age in years (range)**	61.8 (19.7–90.3)	66.4 (45.8–90.3)	72.6 (50.9–81.5)
**FIGO Stage**			
**I**	4	2	4 (2)
**II**		1	2 (1)
**III**		14 (12)	5
**IV**		10 (8)	8 (6)
**NA**	34 (30)	2 (1)	6 (5)

### Methylation analysis

Tissue samples were processed as described previously, including DNA isolation, bisulfite conversion and CpG sites interrogation using Infinium EPICv1 array (based on human genome 19; hg19 and containing 865859 probes) [4, 15].

Cervical swabs were processed as follows. All fractions from the same patient retrieved from The Danish CancerBiobank were pooled together and pelleted by centrifugation. Total DNA was extracted using with QIAamp DNA Mini kit (QIAGEN GmbH, Hilden, Germany) and quantified by a Qubit^®^ 2.0 Fluorometer with Qubit™ dsDNA HS Assay. DNA was bisulfite converted (250 ng per sample) using EZ DNA methylation kit (Zymo Research, Irvine, CA).

Then, samples were subjected to methylation analysis on Infinium EPICv2 array (based on hg38, containing 930075 probes). Methylation analysis of cervical swabs was processed analogously to tissue samples. Briefly, raw data as.idat files were parsed into R using minfi package [16] and processed as described by [17]. Normalization was conducted using functional normalization algorithm (preprocessFunnorm) [18]. Quality assessment was based on the median signal intensity of both methylated and unmethylated probes [16]. Samples with median log_2_ values below 10.5 were discarded/removed due to low quality (altogether 15 out of 92; see Table 1). Low quality probes and probes localizing to common SNPs were filtered out [17].

We used both, beta values and M-values, depending on the type of analysis. Beta values have more intuitive biological interpretation as they can be understood as percentage methylation of a given CpG site. On the other hand, Du et al. recommend using M-values for most statistical analysis, as M-value method has better detection power. It is especially true for the low and high methylation ranges, where beta-values are significantly compressed [19]. Thus, we used beta values for analysis of the relation between samples (principal component analysis; PCA), for sample deconvolution and to calculate WID-OC. M-values were used for differential methylation analysis to pinpoint CpG sites which differ between groups.

### Cell type composition

To estimate proportions of various cell types in biological samples, we used MethylCIBERSORT [20, 21] with extended OC signature as described earlier [15].

### Devising a diagnostic signature

Following sample processing and probe filtration, for each of the 77 cervical samples we obtained information about methylation levels for 852,588 CpG sites. This number was too high to build a robust diagnostic model. Thus, we used several approaches to shortlist the targets/CpG sites and design a model.

#### Splitting samples into training and testing groups

To check which of the models would be stable, we validated them in independent cohorts. Benign/borderline and HGSOC samples were randomly divided into training and testing set, in a ratio 7:3, to obtain altogether ten training– test combinations, separately in the tissue and cervical swab cohorts. Tumor samples categorized as “Other” were added to the testing cohort, in order to see how robust the predictive algorithms would be towards cancers of different histology. Borderline samples, along with benign cases, constituted the control group. Borderline tumors are characterized by low malignant potential and, epigenetically, they more closely resemble benign samples than HGSOC samples [4]. However, they often require surgical intervention. Therefore, if the proposed method proves effective, developing a separate protocol specifically for identifying borderline tumors as a distinct category should be considered.

#### Strategies for candidate selection

This type of analysis was done using M-values. The following strategies were used for shortlisting methylation targets to be then included in a multivariate model:

- **Based on the three-step algorithm.** This process consisted of three steps: (i) applying a univariate linear regression with an empirical Bayes approach [22] to all probes that passed quality control, (ii) submitting the selected probes to a general linear model for further shortlisting via logistic regression, and (iii) applying a LASSO-penalized model for multivariate linear regression to the shortlisted predictors as described in [4].
- **Based on variance.** We compared variance between benign/borderline and HGSOC groups using Bartlett’s test, selected CpG sites with FDR < 0.05 (different variance CpGs, DVC) and further identified CpG sites for which the ratio of variance between HGSOC and benign samples was at least 50. These targets were used to construct a multivariate regression model.

### Estimating signature performance

Performance of a given signature, devised using a training set, was then evaluated in a testing set. Briefly, an index (Y) for each sample from the testing set was calculated using formula of the regression model obtained in the training set as follows:

$${\rm{Y}}\,{\rm{ = }}\,{{\rm{\beta }}_{\rm{0}}}\,{\rm{ + }}\,{{\rm{\beta }}_{\rm{1}}}{{\rm{M}}_{\rm{1}}}\,{\rm{ + }}\,{{\rm{\beta }}_{\rm{2}}}{{\rm{M}}_{\rm{2}}}\,{\rm{ + }}\,{\rm{ \ldots }}\,\,{\rm{ + }}\,{{\rm{\beta }}_{\rm{i}}}{{\rm{M}}_{\rm{i}}} $$

where β_0_ is the intercept, M_1_, M_2_,…, M_i_– M-values for each CpG included in the model and β_1_, β_2_,…, β_i_– coefficients calculated for each CpG included in the model.

Performance of a model was estimated by (i) calculating differences between indexes obtained for samples from benign/borderline versus HGSOC and/or other group and as (ii) the area under the receiver operating characteristic curve (ROC-AUC), predicting if a given sample from the testing cohort is correctly classified.

### Women’s risk IDentification for OC (WID-OC)

WID-OC index was calculated in cervical samples using beta values as described by [14], using the code provided in github.com/IfWH-DoWC/OC-index.

### Statistical analysis

All statistical analyses, including dimensional reduction analysis (PCA), running algorithm for differential methylation analysis, and calculating ROC-AUC, as well as plot preparation were performed in R programming language (version 4.2).

## Results

**Fig. 1:**
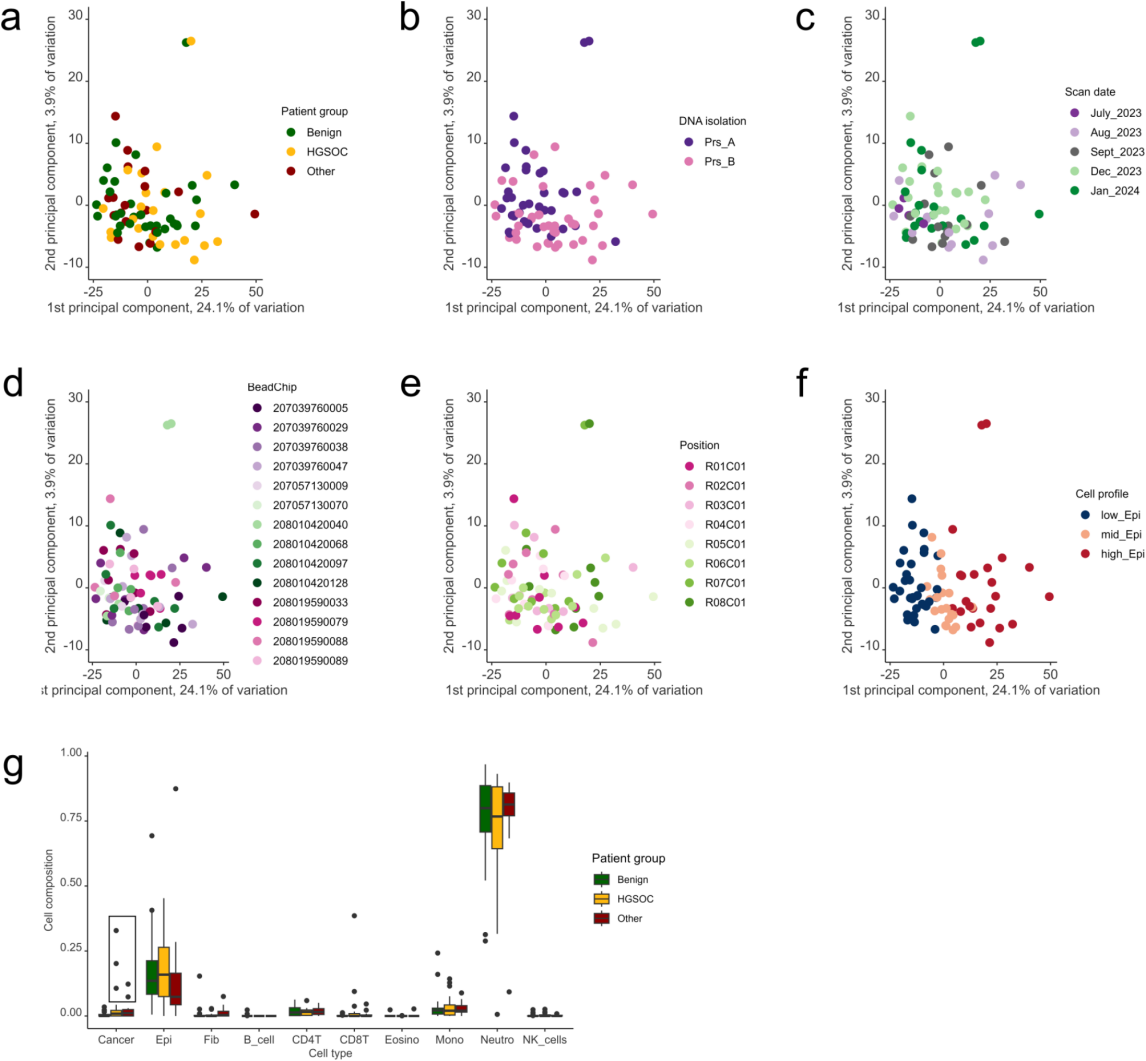
Characteristics of the cervical swabs used in the current study (**a-f**)– principal component analysis for the top 1000 most variable CpG showing grouping of samples depending on different variables; top row from the left: **(a)** patient group (benign, HGSOC or other), **(b)** operator (person responsible for DNA isolation), **(c)** scan date; bottom row from the left: **(d)** BeadChip, **(e)** position on the BeadChip, **(f)** cell composition (low_Epi– content of cells recognized as epithelial, i.e. normal epithelium and cancer together, is below 10%; mid_Epi– between 10 and 20% cells recognized as epithelial; high_Epi– over 20% of cells recognized as epithelial) **(g)**– cell composition as estimated by MethylCIBERSORT across patient groups; black rectancle shows samples with high proportion (over 5%) of cancer cells

Initially, we analyzed similarities within the cohort of cervical swabs. Previously, we observed that ovarian tissue samples tend to cluster by patient group even before implementing any statistical procedures [4]. However, it was not the case in cervical swabs (Fig. 1a). PCA analysis of the top 1000 most variable CpG sites revealed no such tendency among cervical swabs. Then, we set to rule out if it may be due to some technical factors (batch effect) overshadowing biological differences. We tested whether the samples would cluster by the operator (person responsible for DNA isolation), scan date, BeadChip itself or position on the BeadChip, but we observed no tendencies, apart from two samples, localized in neighboring positions on the same BeadChip which could have been outliers (Fig. b-e). Overall, we did not find likely that a batch effect would mask true diagnostic outcome. Eventually, we tested whether the samples would group by cell type profile and obtained a very clear tendency to group according to the proportion of epithelial cells to immune cells (Fig. 1f). A subsequent analysis of cell proportions revealed that cell profiles were similar across patient groups (Fig. 1g). We also noticed a very low proportion of fraction recognized as cancer cells. Cancer cells in amounts exceeding 5% were detected only in 5 samples, and as expected none of these samples belonged to benign group (Fig. 1g).

To identify cancer-specific targets in cervical swabs, we adopted a strategy described by [4]. It has previously been demonstrated that methylation profiles of OC differ from healthy controls or even from benign ovarian disease [4, 23, 24]. Thus, for comparative purposes, we first ran the analysis on a set of ovarian tissue samples. This allowed us to demonstrate the statistical methods’ ability to detect differences between sample groups and illustrate their effectiveness in achieving reliable results The benchmarking ovarian set consisted of 70 samples: benign/borderline (*n* = 17), HGSOC (*n* = 37) and a heterogenous group referred to as “Other” which included ovarian cancer subtypes other than HGSOC as well as cancers which were not primarily ovarian (*n* = 16). The third group (“other”) were there to evaluate robustness of the developed signature, but those samples were not used for developing the signature itself. Benign and borderline cases were included in one category later referred to as “benign”.

**Fig. 2:**
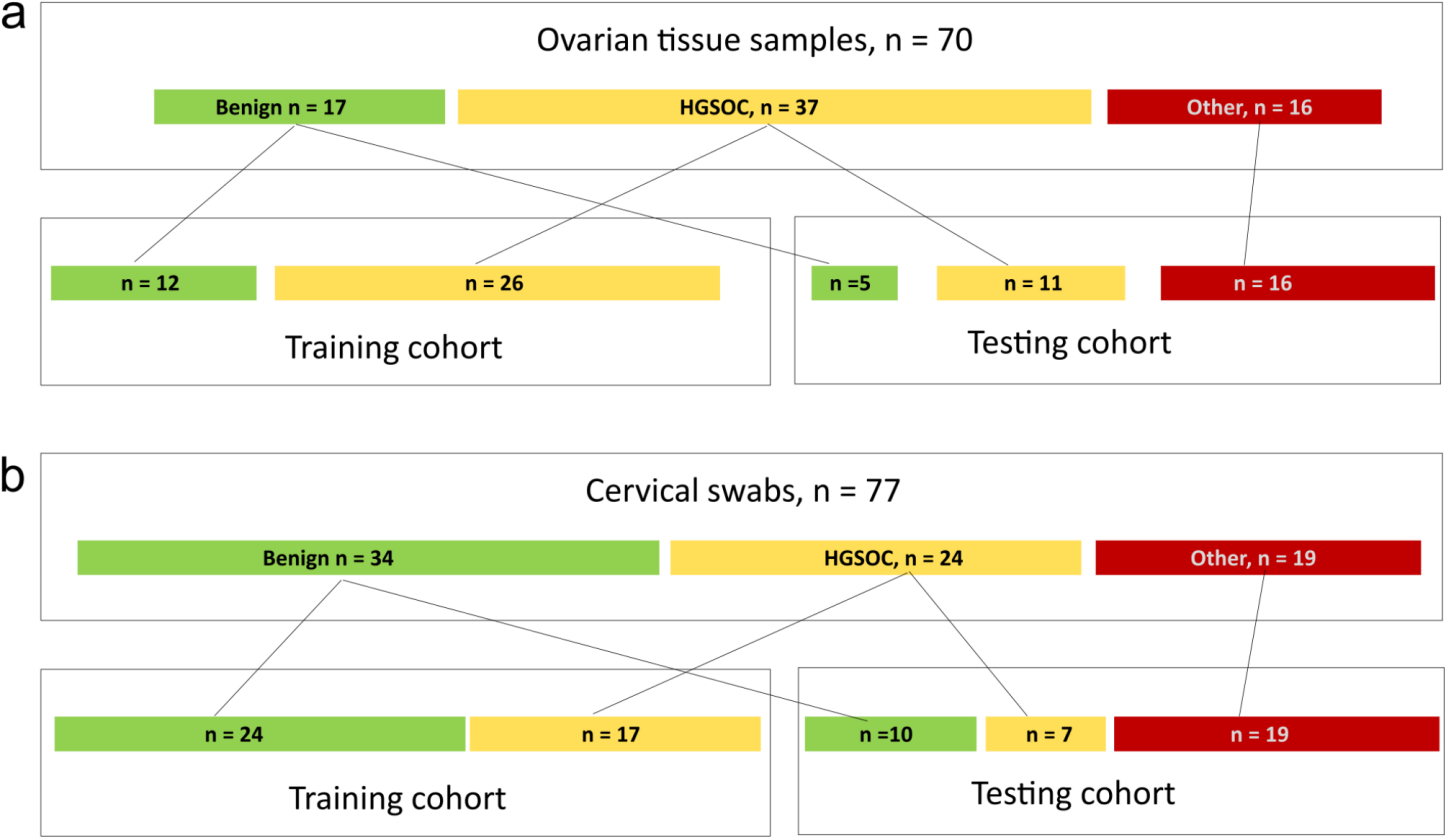
Scheme showing the first split of both cohorts into training and testing sets. **(a)** ovarian cancer tissue samples **(b)** cervical swabs

To start with, we divided the benign and HGSOC samples (17 + 37 = 54) into training and testing set in the proportions 7:3. Thirty-eight samples were used to construct a training set. Sixteen remaining benign/borderline and HGSOC samples along with all “Other” samples were used for the testing set (16 + 16 = 32; Fig. 2a).

**Fig. 3:**
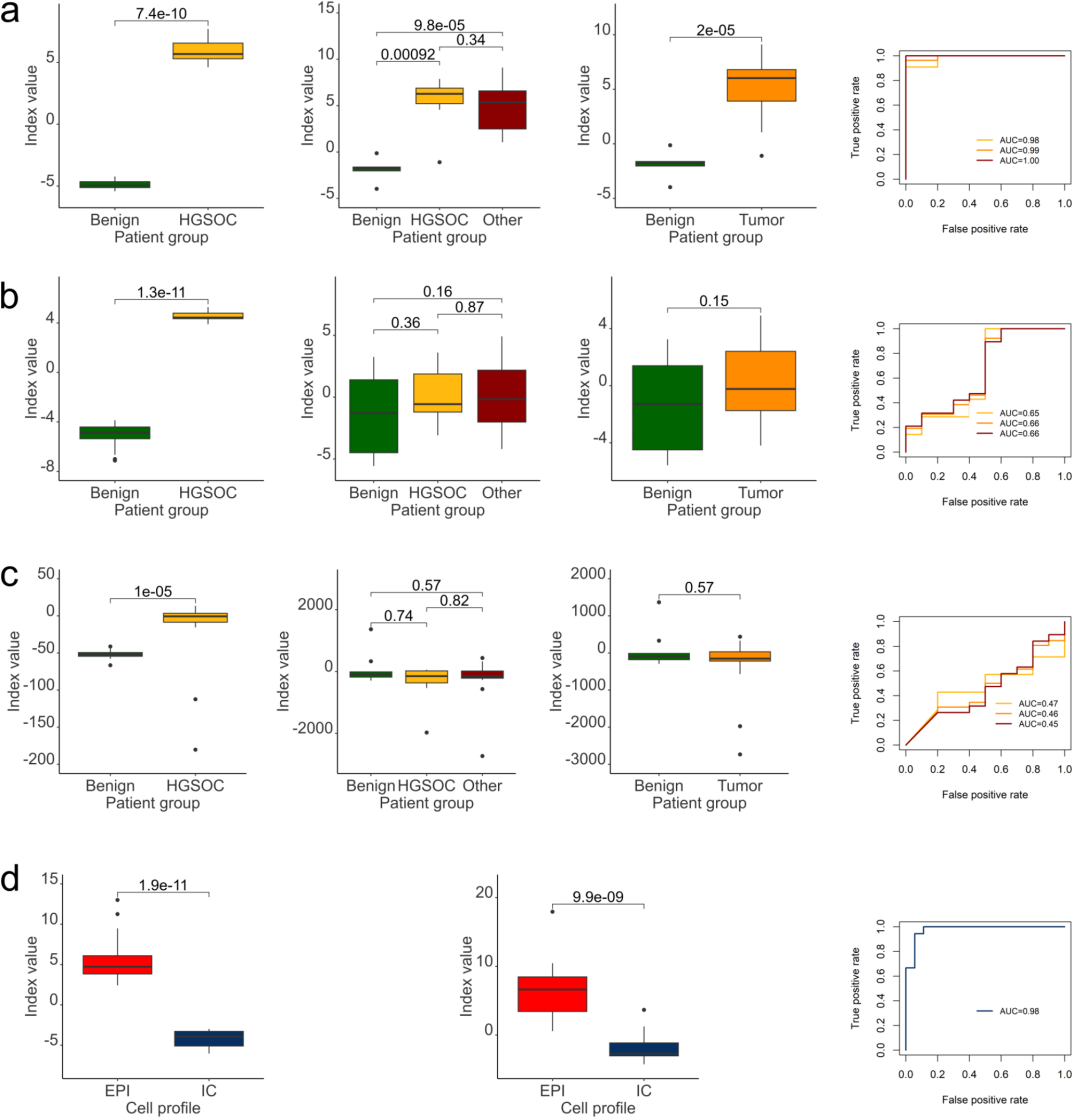
Identification of CpG sites that enable classification of samples into groups. Graphical illustration of the analyses performed for split 1; green color refers to benign samples, yellow– HGSOC, dark red– ”other” group, orange– all tumor samples pooled together (HGSOC + other), light red– samples with high epithelial content, dark blue– samples with high immune content **(a)** ability of the three-step algorithm to classify ovarian tissue samples into benign and malignant; from left to right: performance of the model in the training set shown as index values, performance of the model in the testing set, separately for HGSOC and ”other”; performance of the model in the testing set, but showing all tumor samples together (HGSOC + other); performance of the model in the testing set for each group shown as ROC-AUC **(b)** ability of the three-step algorithm to classify cervical swabs into benign and malignant; set of sub-panels analogous to a); **(c)** ability of the model constructed basing on variance ratio to classify cervical swabs into benign and malignant; set of sub-panels analogous to a); **(d)** ability of the three-step algorithm to classify cervical swabs into high and low epithelial content; from left to right: performance of the model in the training set shown as index values, performance of the model in the testing set, performance of the model in the testing set shown as ROC-AUC

Outcome of each step of the selection strategy can be found in Supplementary Table S1. Briefly, the first step (univariate linear model) resulted in 140,940 targets which methylation levels significantly differed (adjusted p-value < 0.05) between benign and HGSOC groups. Of those, 31,950 (adj. p-value < 0.05 and logFC >|2|) were subjected to univariate logistic regression which further identified 6,753 targets. The shortlisted targets were subjected to a LASSO-penalized multivariate logistic regression, resulting in a model containing 25 targets. Model coefficients and intercept were then applied to calculate index values for each sample (see Methods). These index values were subsequently used to validate the model with the testing set. Sensitivity and specificity of the model were estimated using ROC-AUC (Supplementary Table S1 and Fig. 3a) in three different approaches: benign vs. HGSOC, benign vs. all tumor cases together and benign vs. “other”. In all cases, the selected 25 targets allowed very good discrimination between malignant and benign cases. The whole statistical procedure was then repeated nine times (for the total of ten splits into training and testing cohort), with a comparable outcome (Supplementary Table S1).

Subsequently, we attempted a similar strategy using cervical swabs. Sample cohort was again divided into training (*n* = 41) and testing (*n* = 36) sets (Fig. 2b). When running the algorithm, already the first step had an unexpected result: after correcting for multiple comparisons, none of the 852,588 targets was significantly different between benign and HGSOC group. Moreover, only very few targets showed difference between both groups which would exceed logFC >|2|. Thereby we used relaxed selection criteria for the following steps and allowed 7,822 targets for which unadjusted p-value < 0.05 and logFC >|0.5|. The resulting model (also consisting of 25 targets, though not overlapping with those identified in the tissue) successfully separated samples from the training set, but performed very poorly in the independent, testing set (AUC around 0.65; Fig. 3b and Supplementary Table S2).

To exclude that the results obtained were due to random separation into training and testing sets, we ran the analysis, like we did for tissue samples, nine times more (for the total of ten splits). Results of the repeated analysis were largely similar to the first run (see Supplementary Table S2).

We also considered a different approach of identifying potential candidates for development of a diagnostic signature. We reasoned that while non-neoplastic samples may exhibit a rather similar, consistent methylation landscape, a malignant phenotype may be more heterogenous. In such case, cancer-specific methylation would manifest as “outliers” or atypically methylated sites, but perhaps with low frequency. Such features, even if typical of cancer, may not be caught by statistical tests like t-test. However, they may reflect in sample variance, as also postulated by Teschendorff and coworkers ( [25]; see Discussion). Therefore, when constructing a multivariate model, we adopted another shortlisting strategy, which took under consideration differences between variance of M-values between benign and malignant samples. Briefly, we selected targets where the ratio between variance of malignant samples to variance of benign samples was at least 50. Targets selected this way were then used as input for multivariate logistic regression. However, obtained models also performed very poorly: in most cases no better than a random guess and for some of the splits the AUC was even below 0.5 (Fig. 3c and Supplementary Table S3). Perhaps that should not come as a surprise, since (unlike in ovarian tissue samples) the differences in the variance were only driven by single outliers (Supplementary Fig. S1).

Above findings suggested that identifying targets with diagnostic potential using cervical swabs as a surrogate material may pose a challenge. We speculated that it may be due to other factors, overshadowing differences resulting from malignant transformation. PCA analysis performed as a part of the technical quality check showed that samples did not cluster according to any of the technical variables. However, they did cluster depending on the proportion of the epithelial to immune cells. Therefore, we divided samples into low (below 10%) and high immune content. Interestingly, we were not only able to identify differentially methylated probes between both groups of samples, but also the developed model could be successfully used to predict to which group samples from the testing cohort would belong (AUC > 0.95 in all 10 cases; Fig. 3d and Supplementary Table S4).

**Fig. 4:**
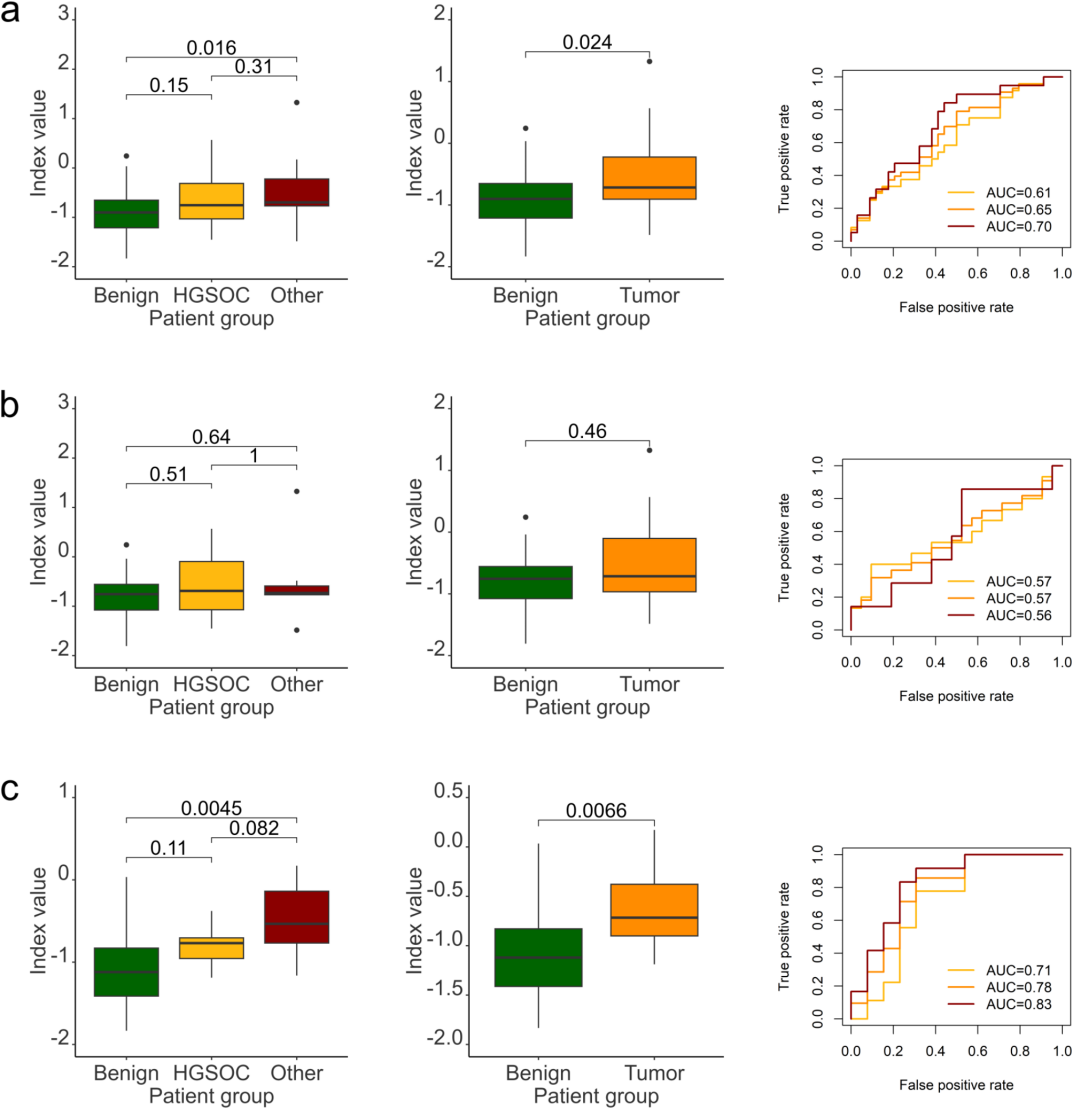
Performance of WID-OC in the study cohort of cervcal swabs. Indexes are calculated as beta values **a)** performance of WID-OC in the whole cohort, irrespective of cell composition; from left to right: differences between WID-OC index between benign versus HGSOC and versus ”other” groups separately; benign versus all tumors pooled together (HGSOC + other); performance of WID-OC for each group shown as ROC-AUC **b)** performance of WID-OC in 43 samples with high epithelial content; set of sub-panels analogous to **a**) **c)** performance of WID-OC in 34 samples with high immune content; set of sub-panels analogous to **a**)

Then, we validated the WID-OC index, originally developed by Barrett et al. (2022) to identify women at risk of ovarian cancer (OC) [14]. This index incorporates beta-values from 14,000 CpGs. Due to probe filtering (e.g., removal of poorly performing probes, as described in Methods) and differences between array platforms (EPICv1 vs. EPICv2), we only included 12,284 CpG. Using all 77 cervical smear samples, the index differentiated between benign and “other” samples, and between benign and all cancers combined, but not between benign and HGSOC (Fig. 4a). This suggests that the mean index value was elevated due to the “other” group. In a real-world scenario, where HGSOC accounts for approximately 70% of cases, this would result in many missed diagnoses. Interestingly, the highest AUC was observed in the “other” group, a heterogeneous set of patients (AUC = 0.7).

We also tested performance of WID-OC separately in samples with high and low content of immune cells. Interestingly, WID-OC performed better in samples with high content of immune cells, unlike what the authors originally observed [14]. Again, WID-OC performed best in samples belonging to the class “other”, with AUC = 0.83 (Fig. 4b and c).

**Fig. 5:**
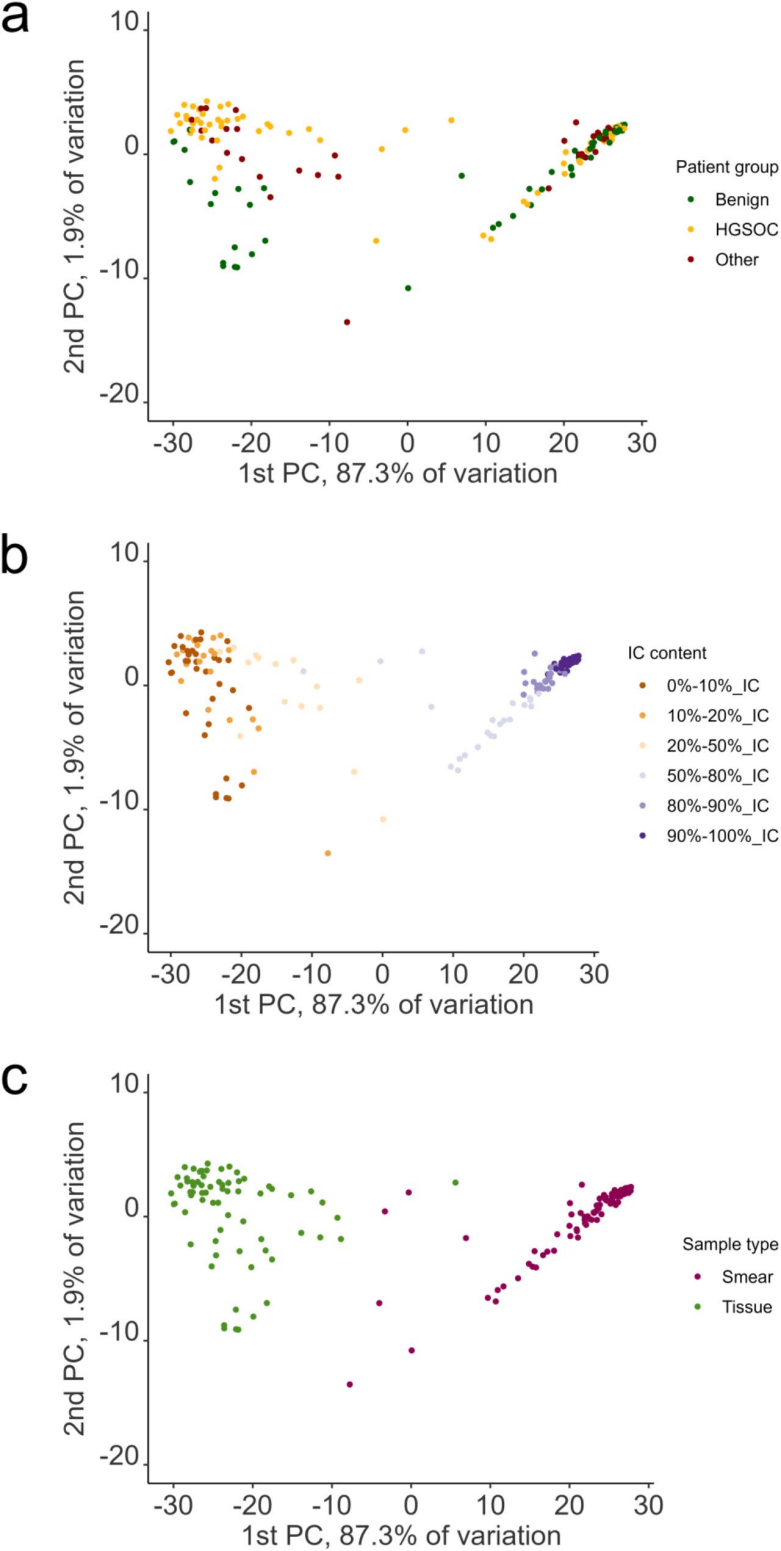
Principal component analysis showing similarity between both cohorts– ovarian tissue samples and cervcal swabs– together. Top 5000 most variable CpGs were used for the analysis. Sample grouping is shown based on **(a)** patient group **(b)** cell composition **(c)** source of material– ovarian tissue versus cervical swab

Eventually, we analyzed similarity between ovarian tissue samples and cervical swabs and performed PCA on samples from both cohorts (70 ovarian tissue samples and 77 cervical swabs). The picture we saw was highly consistent with the above findings (Fig. 5). Ovarian tissue samples presented a heterogenous group, and even without any filtration or selection criteria, samples from patients diagnosed with benign ovarian tumor tended to group together (Fig. 5a). At the same time, cervical swabs presented a rather compact cluster by themselves, with much smaller spread than in case of tissue samples. As already seen in Fig. 1a no clustering based on diagnosis was visible. Unlike cervical swabs, clustering of ovarian tissue samples was not affected by the proportion of immune cells (Fig. 5b). In the analysis presented, however, samples clearly separated based on the source of material (tissue versus swab) (Fig. 5c).

## Discussion

An optimal diagnostic screening tool should be affordable, practical, and use easily accessible biological material. A PCR or sequencing panel with DNA isolated from cervical swabs would meet these criteria. Here, we exploited the possibility to use cervical swabs for early diagnosis of OC. To select promising CpG scandidates, we interrogated methylation profiles of patients with malignant and benign pelvic masses using Infinium EPICv2 array.

We noticed that cervical swabs were a more challenging material than frozen tissue samples. A higher number of cervical swabs failed quality check compared to fresh frozen tissue samples (15 out of 92 i.e., 16% and 2 out of 72 i.e., 2.7% respectively). However, this difference may not only result from the quality and integrity of the genomic material, but also the DNA input (500 ng versus 250 ng DNA per sample for tissue samples and cervical swabs respectively). Lowering the DNA was necessary, because of the overall lower DNA yield obtained from cervical swabs. Still, sample quality was in most cases sufficient to get the relevant information and our earlier study suggests that cervical swabs could be successfully interrogated by sequencing panels (manuscript under revision).

Our main goal was to check whether the extent of differences seen between cervical swabs obtained from patients with benign and malignant pelvic mass warrants their use as a diagnostic tool. However, when constructing a diagnostic model, we observed that, after correcting for multiple comparisons, none of the CpG sites was differentially methylated between cervical swabs taken from benign and HGSOC patients. A similar observation was previously made by [25]. They postulated that ordinary t-tests and their non-parametric equivalents may be underpowered to detect methylation changes in preneoplastic samples or in normal tissue adjacent to cancer tissue. However, they argued, a range of CpG sites in such samples would show variance different than in their normal counterparts [25]. Further, they identified three types of such differentially variable CpGs (DVC). In case of those belonging to the first type, both the variance and the mean methylation level would differ between phenotypes of interest (e.g., normal and premalignant). In type 2 and type 3 mean methylation levels between phenotypes are similar and statistically undistinguishable. However, in type 2 the differences in variance are driven just by few outliers (few samples), usually showing unidirectional changes (hyper- or hypomethylation), while in type 3 by higher number of samples, showing changes in both directions. In our cohort, in cervical swabs, differences in variance were driven only by few isolated outliers (type 2), and in some cases (for example cg22839417) were not reproduced in the test cohort. As expected, methylation changes driving differential variation in ovarian tissue samples, showed higher frequency rate (type 1 and 3). Thus, despite changing the strategy of targets selection and accounting for the possible low frequency of methylation changes, we were not able to identify CpG sites discriminating between benign and malignant phenotype in cervical swabs.

We then checked how a previously published index (WID-OC index) would perform in our cohort [14]. Albeit we could see that the malignant samples generally scored higher than benign, the specificity and sensitivity is, according to our findings, not sufficient to use WID-OC in clinical practice. Moreover, it was built basing on as many as 14,000 CpG sites, of which 12,284 overlapped with CpG sites present in our dataset.

WID-OC was constructed with an assumption that changes between cervical swabs from benign and malignant ovarian patients are not driven by the presence of tumor cells, but by the overall differences in the methylation landscape. In agreement with their findings, we also observed that presence of tumor DNA was detectable in the cervical swabs only in very few cases (5 out of 43 malignant cases), as inferred computationally through deconvolution of methylation profiles. Moreover, we also noticed that the two dominant cell types are epithelial cells and neutrophils, even though we used different deconvolution methods, namely HEpiDISH and MethylCIBERSORT, which differed both by the reference panel and computational algorithm [20, 26]. The proportion of immune cells seem very high in cervical swabs. However, it is a question of the proportions of the genetic material of certain source rather than cell type proportion per mass or per volume. This can be much lower than suggested by the deconvolution results as epithelial cells are typically about twice larger than neutrophils.

Analysis of the similarity between the samples (PCA) indicated that cervical swabs mainly grouped according to cell type composition while tissue samples showed a tendency to cluster according to patient group. This aligns with the findings of Qi and Teschendorff, who identified factors contributing to the main sources of variation among samples. They demonstrated that cell-type heterogeneity generally has a greater impact than sex, age, or ethnicity. However, differences between normal and cancerous tissues are often linked to substantial global changes in the methylation landscape, which account for the top components of variation alongside cell-type heterogeneity [27].

Global methylation changes in tumor tissue readily distinguish tumor samples from those of patients with benign disease; however, these changes are not mirrored in the methylation profiles of cervical swabs. This study explores an approach for early ovarian cancer detection. Of note, among the 29 HGSOC patients included for cervical swab analysis, only 3 had stage I or II cancers. Despite the high prevalence of advanced-stage cancers in the cohort, the study did not identify reliable markers, further supporting the conclusion that local methylation profiles alone are insufficient for diagnostic purposes.

## Conclusions and future perspectives

In the light of the above findings, both the results of differential methylation analysis and the PCA, challenges related to developing a stable diagnostic signature seem understandable. It seems that basing on the overall methylation landscape of cervical swabs may not suffice to design tools for early detection of OC. However, an approach previously suggested by several groups who postulated that cancer DNA can be found in the cervical swabs, may still be worth pursuing. So far sensitivity of OC detection was estimated to be around 40% [5], but hopefully, with identifying the right targets and implementing more sensitive methods, this number can be increased, allowing detection of cancer-specific motifs even in samples with low tumor content. Our study primarily presents negative findings. However, we believe these are also important to share, as they help prevent research redundancy, reduce publication bias, and guide future studies toward more promising diagnostic approaches.

## Electronic supplementary material

Below is the link to the electronic supplementary material.

Supplementary Material 1

Supplementary Material 2

Supplementary Material 3

## Data Availability

No datasets were generated or analysed during the current study.
