# Supplementary material for: Potential use of DNA methylation in cervical swabs for early ovarian cancer diagnosis
Source: J Ovarian Res. 2025 Feb 15;18:29. doi: 10.1186/s13048-025-01609-2 (PMC11830180; doi:10.1186/s13048-025-01609-2)
Supplement: Supplementary file 2 — Supplementary Material 2 [file 13048_2025_1609_MOESM2_ESM.docx]

**Potential use of DNA methylation in cervical swabs for early ovarian cancer diagnosis**

Edyta Biskup ^1^, **Joanna Lopacinska-Jørgensen ^1^,** Claus Høgdall ^2^, Estrid V. Høgdall ^1^

**^1^** Department of Pathology, Herlev Hospital, University of Copenhagen, Herlev, Denmark

^2^ Department of Gynaecology, Juliane Marie Centre, Rigshospitalet, University of Copenhagen, Copenhagen, Denmark

**Supplementary figure S1.Distribution of beta values of CpG sites showing the highest ratio of variance between tumor and benign samples.** Training and testing sets refer to split 1. **a)** top three CpG sites characteristic for cervical swabs in training set of cervical swabs **b)** the same CpG sites in testing set of cervical swabs **c)** top three CpG sites characteristic for ovarian tissue samples in training set of ovarian tissue samples **d)** the same CpG sites in testing set of ovarian tissue samples
