# Supplementary figures and images for: Potential use of DNA methylation in cervical swabs for early ovarian cancer diagnosis
Source: J Ovarian Res. 2025 Feb 15;18:29. doi: 10.1186/s13048-025-01609-2 (PMC11830180; doi:10.1186/s13048-025-01609-2)

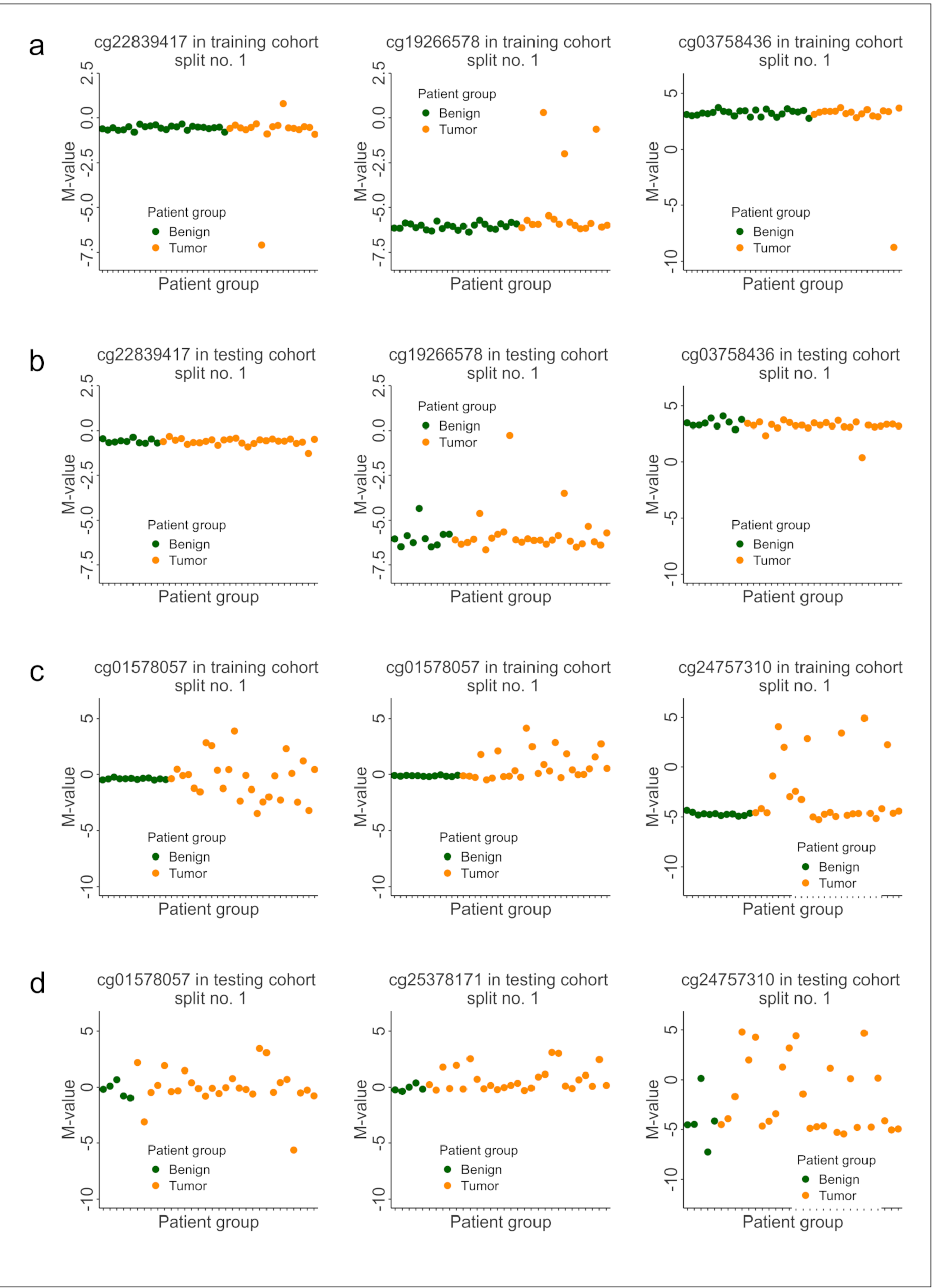

Supplement: Supplementary file 3 — Supplementary Material 3 [file 13048_2025_1609_MOESM3_ESM.tiff]
